# RAPD and Internal Transcribed Spacer Sequence Analyses Reveal *Zea nicaraguensis* as a Section *Luxuriantes* Species Close to *Zea luxurians*

**DOI:** 10.1371/journal.pone.0016728

**Published:** 2011-04-15

**Authors:** Pei Wang, Yanli Lu, Mingmin Zheng, Tingzhao Rong, Qilin Tang

**Affiliations:** Maize Research Institute, Sichuan Agricultural University, Ya'an, Sichuan, China; St. Petersburg Pasteur Institute, Russian Federation

## Abstract

Genetic relationship of a newly discovered teosinte from Nicaragua, *Zea nicaraguensis* with waterlogging tolerance, was determined based on randomly amplified polymorphic DNA (RAPD) markers and the internal transcribed spacer (ITS) sequences of nuclear ribosomal DNA using 14 accessions from *Zea* species. RAPD analysis showed that a total of 5,303 fragments were produced by 136 random decamer primers, of which 84.86% bands were polymorphic. RAPD-based UPGMA analysis demonstrated that the genus *Zea* can be divided into section *Luxuriantes* including *Zea diploperennis*, *Zea luxurians*, *Zea perennis* and *Zea nicaraguensis*, and section *Zea* including *Zea mays* ssp. *mexicana*, *Zea mays* ssp. *parviglumis*, *Zea mays* ssp. *huehuetenangensis* and *Zea mays* ssp. *mays*. ITS sequence analysis showed the lengths of the entire ITS region of the 14 taxa in *Zea* varied from 597 to 605 bp. The average GC content was 67.8%. In addition to the insertion/deletions, 78 variable sites were recorded in the total ITS region with 47 in ITS1, 5 in 5.8S, and 26 in ITS2. Sequences of these taxa were analyzed with neighbor-joining (NJ) and maximum parsimony (MP) methods to construct the phylogenetic trees, selecting *Tripsacum dactyloides* L. as the outgroup. The phylogenetic relationships of *Zea* species inferred from the ITS sequences are highly concordant with the RAPD evidence that resolved two major subgenus clades. Both RAPD and ITS sequence analyses indicate that *Zea nicaraguensis* is more closely related to *Zea luxurians* than the other teosintes and cultivated maize, which should be regarded as a section *Luxuriantes* species.

## Introduction

The genus *Zea* has been classified into two sections [1], [2], section *Luxuriantes*, which is composed of *Zea diploperennis*, *Zea luxurians* and *Zea perennis*, and section *Zea*, which contains four subspecies: *Zea mays* ssp. *mays*, *Zea mays* ssp. *mexicana*, *Zea mays* ssp. *parviglumis* and *Zea mays* ssp. *huehuetenangensis* [3]. In genus *Zea*, both wild taxa that have the common name “teosinte” and cultivated maize are diploid (n = 10) with the exception of tetraploid *Z. perennis* (n = 20). As the closest wild relative of maize, teosinte, which is indigenous to Mexico and Central America [4], is a potentially important resource for the study of maize genetics and evolution and for plant breeding.

A new teosinte recently discovered from Pacific Coastal Nicaragua, named *Zea nicaraguensis*, occurs at 6–15 m above sea level, a very low elevation for teosinte, and has the unusual ability to grow in as much as 0.4 m of standing or slowly moving water [5]. Thriving in flooded conditions, this fresh teosinte has a high capacity to form root aerenchyma and adventitious roots. *Z. nicaraguensis*, noteworthy for its tolerance to a waterlogged environment and for its stable expression of a reproductive pathway [6], could be a useful source of germplasm for maize breeding, specifically, for breeding flooding-tolerant maize accessions through wide cross [5], [7]. The closest relative to Nicaraguan teosinte, *Z. nicaraguensis*, is probably *Z. luxurians* of southeastern Guatemala [5]. *Z. nicaraguensis* and *Z. luxurians* show close morphological resemblance, and also display considerable differences in developmental behaviour, supporting a taxonomic segregation [8]. Nicaraguan teosinte has much longer and more abundant tassel branches, a larger number of spikelets per branch, and longer, more visibly transversely rugose outer glumers, as well as a habitat different from its Guatemalan counterpart [5]. However, the actual genetic relationship of *Z. nicaraguensis* in *Zea* species is uncertain until now. Iltis and Benz [5] considered *Z. nicaraguensis* as a new species based on differences in ecology and tassel and plant morphology. The chromosome number of *Z. nicaraguensis* is 2n = 20, and the C-banding pattern shows that *Z. nicaraguensis* is very similar to *Z. luxurians* and more similar to *Z. luxurians* than to *Z. diploperennis* and cultivated maize [8]. Nevertheless, the genetic relationship of *Z. nicaraguensis* in *Zea* species should be further investigated systematically.

Recently-developed molecular genetic techniques have provided another opportunity to assess the degree of genetic relatedness between maize and teosinte. RAPD marker has proven quite useful in genetic study of many plant species [9]. This marker system has the ability to amplify DNA from dispersed polymorphic loci and has its power to detect small genetic differences [10]. To our knowledge, such molecular marker is only an indirect DNA sequence analysis technique. To infer a more accurate conclusion on genetic relationships among species, it is necessary to combine with direct DNA sequence analysis techniques. Previous studies on the internal transcribed spacer (ITS) region of the rDNA in nuclear genome showed promising results for the phylogenetic study of grasses [11], [12]. We have chosen ITS as a phylogenetic marker, because it is appropriate for investigating species-level relationships and it is a nuclear marker that can be useful for detecting reticulate phenomena [13]. ITS sequences evolve rapidly, but size and functional constraints permit comparison of homologous sequences between taxa in genus or subgenus [14].

In this report, we present the first molecular marker analyses for the genetic relationship of *Z. nicaraguensis* in *Zea* species by using randomly amplified polymorphic DNA (RAPD) markers and the internal transcribed spacer (ITS) sequences of nuclear ribosomal DNA (nrDNA), which have both been used to resolve relationships among closely related taxa [15], and a sample of 14 maize and teosinte species. Comparisons between clustering trees inferred from both indirect and direct DNA sequence analysis techniques would provide a better assessment of the true species relationships.

## Results

### RAPD band polymorphism

**Figure 1:**
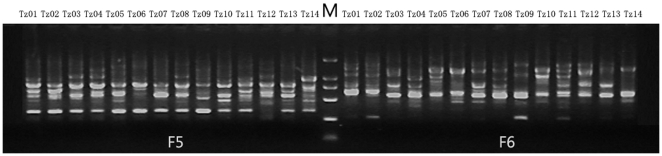
Banding patterns of RAPD produced by the primers F5 and F6. Tz01—*Z. perennis*, Tz02—*Z. perennis*, Tz03—*Z. mays* ssp. *parviglumis*, Tz04—*Z. mays* ssp. *parviglumis*, Tz05—*Z. luxurians*, Tz06—*Z. luxurians*, Tz07—*Z. mays*. ssp. *mexicana*, Tz08—*Z. mays*. ssp. *mexicana*, Tz09—*Z. diploperennis*, Tz10—*Z. nicaraguensis*, Tz11—*Z. mays*. ssp. *huehuetenangensis*, Tz12, Tz13, Tz14—*Z. mays*. ssp. *mays*. M: Marker, DL 2000.

A total of 4500 polymorphisms out of 5303 repeatable products ranging from 500 to 2000 bp were obtained from 136 decamer primers screened from 340 primers among maize and teosinte species (Figure 1). The ratio of polymorphism was 84.86% and each primer generated an average of 38.99 bands and 33.09 polymorphic bands, indicating extensive genetic diversity existing in *Zea* species.

### Genetic similarities among *Zea* species by RAPD analysis

The RAPD data were used to estimate the genetic similarities and the phylogenetic relationships among the 14 *Zea* species genotypes. A similarity coefficient matrix showed the genetic distance among all *Zea* species in the present study (Table 1). The similarity values ranged from 0.585 to 0.809 with an average similarity value 0.671. For the same species from different geographical regions compared, the highest similarity index (0.809) was observed between the Tz01 and Tz02 genotypes, followed by the similarity index between Tz05 and Tz06 (0.796). For the different species compared, the highest similarity index (0.745) was observed between the Tz04 and Tz07 species, and the lowest one (0.585) was recorded between Tz01 and Tz11. A high degree of genetic similarity was revealed among the same species from different geographical regions. For *Z. nicaraguensis*, the similarity index between Tz10 and Tz06 was the highest (0.736), and the one between Tz10 and Tz02 was the lowest (0.593), indicating the close relationship between *Z. nicaraguensis* and *Z. luxurians*.

**Table 1:** Similarity coefficients of 14 accessions/species of genus *Zea* by using RAPD primers.

	Tz01	Tz02	Tz03	Tz04	Tz05	Tz06	Tz07	Tz08	Tz09	Tz10	Tz11	Tz12	Tz13	Tz14
Tz01	1.000													
Tz02	0.809	1.000												
Tz03	0.635	0.647	1.000											
Tz04	0.624	0.638	0.767	1.000										
Tz05	0.644	0.641	0.646	0.635	1.000									
Tz06	0.621	0.612	0.624	0.610	0.796	1.000								
Tz07	0.629	0.644	0.737	0.745	0.628	0.651	1.000							
Tz08	0.603	0.601	0.699	0.706	0.635	0.617	0.784	1.000						
Tz09	0.695	0.696	0.610	0.633	0.635	0.612	0.650	0.638	1.000					
Tz10	0.626	0.593	0.611	0.604	0.733	0.736	0.621	0.620	0.639	1.000				
Tz11	0.585	0.588	0.651	0.664	0.613	0.607	0.682	0.692	0.621	0.644	1.000			
Tz12	0.598	0.602	0.662	0.674	0.639	0.631	0.689	0.680	0.594	0.631	0.682	1.000		
Tz13	0.594	0.601	0.683	0.695	0.601	0.617	0.700	0.687	0.586	0.608	0.670	0.808	1.000	
Tz14	0.607	0.603	0.674	0.686	0.625	0.625	0.693	0.686	0.595	0.639	0.653	0.759	0.787	1.000

### RAPD cluster analysis

**Figure 2:**
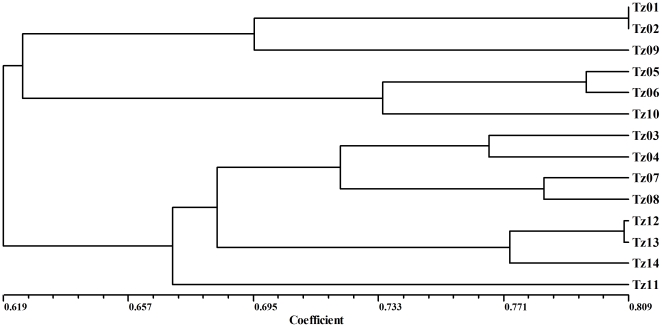
A dendrogram revealed by cluster analysis using RAPD markers. The scale indicates the coefficient of similarity among the 14 species/accessions of *Zea*.

The similarity matrix was then used to construct a dendrogram which showed two major clusters within 14 species of *Zea* at the level of 0.619 with the UPGMA (unweighted pair group method with arithmetic average) method (Figure 2). The first major cluster included *Z. diploperennis* (Tz09), *Z. perennis* (Tz01 and Tz02), *Z. luxurians* (Tz05 and Tz06) and *Z. nicaraguensis* (Tz10). The second major cluster consisted of *Z. mays* ssp. *parviglumis* (Tz03 and Tz04), *Z. mays* ssp. *mexicana* (Tz07 and Tz08), *Z. mays* ssp. *huehuetenangensis* (Tz11) and *Z. mays* ssp. *mays* (Tz12,Tz13 and Tz14). The results were generally consistent with the opinion that the genus *Zea* was divided into sections *Luxuriantes* and *Zea* according to Dobeley et al. [1] and Iltis et al. [2]. The new teosinte *Z. nicaraguensis* was classified into the first major cluster and thus considered to be a section *Luxuriantes* species. It formed a subgroup with *Z. luxurians*, indicating their closest relationship.

Within the two major clusters, the section *Luxuriantes* clade indicated that Z. *diploperennis* and Z. *perennis* formed a subgroup at the level of 0.626, which contained two genotypes only belonging to the perennis tesionte species. The section *Zea* was divided into three subgroups at the level of 0.686, with the first subgroup including *Z. mays* ssp. *parviglumis* and *Z. mays* ssp. *mexicana*, the second subgroup consisting of three cultivated maize inbreds, and the third subgroup only containing *Z. mays* ssp. *huehuetenangensis*.

### ITS sequence variation

**Figure 3:**
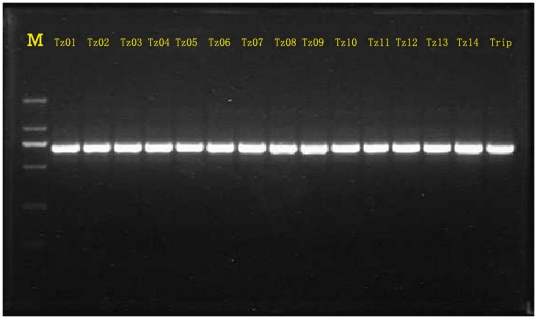
Amplified ITS of the plant materials used in the present study. M: Marker, DL 2000.

The PCR-amplified DNA fragments of all samples showed a clean single band product when examined on an agarose gel (Figure 3).

The boundaries of ITS1, 5.8S and ITS2 regions were identified by comparison with published ITS sequences of maize and teosinte available from GenBank referring to Buckler and Holtsford [16]. The ITS sequences in the present study were highly homologous (95–99%) with *Zea* ITS sequences reported by Buckler et al. [16], demonstrating that our sequences are accurate and reliable. Characteristics of the ITS1, 5.8S and ITS2 sequences of *Zea* species were summarized in Table 2. The lengths of the entire ITS regions of the 14 taxa analyzed varied from 597 to 605 bp. The ITS1 sequences which varied in size from 211 to 218 bp were only slightly shorter than ITS2 (221–229 bp), and the 5.8S gene was uniformly 164 bp in length. The average GC content of the total ITS region was 67.8%; ITS1, 5.8S, and ITS2 regions had average GC contents of 70.4%, 56.6%, and 73.5%, respectively. All of the *Zea* alleles had similar base composition. When the outgroup taxa (*Tripsacum dactyloides* L.) was taken into account, the alignment of the entire ITS sequences resulted in 614 characters, of which 10 positions (of 78 variable sites) were potentially phylogenetically informative sites. Five sites (of 47 variable sites) were informative in ITS1, 5 (of 26 variable sites) in ITS2, and no informative site was recorded in the 5.8S gene which contained 5 variable sites (Table 2).

**Table 2:** Characteristics of the nrDNA ITS sequences in *Zea*.

Sequence characteristics	ITS1	5.8S	ITS2	Total region
Length range (bp)	211–218	164	221–229	597–605
GC content (mean %)	70.4	56.6	73.5	67.8
Length (bp) (aligned)	218	164	232	614
Constant sites	171	159	206	536
Variable sites	47	5	26	78
Informative sites	5	0	5	10

For the combined data set (ITS1, 5.8S, ITS2), pairwise nucleotide sequence divergence based on Kimura two-parameter distance method [17] ranged from 0.17 to 2.92% among taxa of *Zea*, with a mean value of 1.29%; between species of *Zea* and the outgroup taxa, it ranged from 0.17 to 9.54% (Table 3). The divergence values from pairwise comparisons between Tz03 and Tz04, Tz06 and Tz10, and Tz05 and Tz10 genotypes were the lowest, and the greatest divergence was found between Tz02 and Tz13. The sequence divergence between *Z. nicaraguensis* and the other taxa in *Zea* ranged from 0.17 to 2.20%, among which the distances between Tz10 and Tz05, and Tz10 and Tz06 were the lowest, confirming that *Z. nicaraguensis* is more similar to *Z. luxurians* than to the rest wild and domesticated species.

**Table 3:** The distance matrix based on Kimura two-parameter distance method.

	Tz01	Tz02	Tz03	Tz04	Tz05	Tz06	Tz07	Tz08	Tz09	Tz10	Tz11	Tz12	Tz13	Tz14	Trip
Tz01	0.0000														
Tz02	0.0118	0.0000													
Tz03	0.0119	0.0170	0.0000												
Tz04	0.0101	0.0153	0.0017	0.0000											
Tz05	0.0085	0.0136	0.0135	0.0101	0.0000										
Tz06	0.0085	0.0136	0.0118	0.0085	0.0034	0.0000									
Tz07	0.0170	0.0223	0.0101	0.0067	0.0152	0.0153	0.0000								
Tz08	0.0153	0.0205	0.0101	0.0067	0.0135	0.0135	0.0084	0.0000							
Tz09	0.0084	0.0136	0.0135	0.0119	0.0118	0.0118	0.0205	0.0187	0.0000						
Tz10	0.0068	0.0119	0.0118	0.0084	0.0017	0.0017	0.0135	0.0118	0.0101	0.0000					
Tz11	0.0136	0.0188	0.0084	0.0050	0.0169	0.0153	0.0135	0.0135	0.0170	0.0135	0.0000				
Tz12	0.0153	0.0204	0.0067	0.0050	0.0152	0.0135	0.0050	0.0084	0.0169	0.0135	0.0101	0.0000			
Tz13	0.0239	0.0292	0.0203	0.0169	0.0237	0.0238	0.0187	0.0151	0.0274	0.0220	0.0237	0.0186	0.0000		
Tz14	0.0136	0.0188	0.0084	0.0050	0.0135	0.0118	0.0033	0.0067	0.0170	0.0101	0.0118	0.0033	0.0169	0.0000	
Trip	0.0805	0.0862	0.0841	0.0806	0.0708	0.0746	0.0879	0.0840	0.0840	0.0727	0.0860	0.0860	0.0954	0.0841	0.0000

Gaps due to insertion/deletion events were introduced to align the sequences of the ITS1 and ITS2 regions. The largest gap (9 bp) was in the ITS2 region of the *T. dactyloides* sequence. Hsiao et al. [12] indicated gaps were correlated with particular species groups and were potentially phylogenetically informative. Baldwin [18] also suggested that length mutation in the ITS region can be of potential value for phylogeny reconstruction.

In addition to the insertion/deletions, the most common polymorphisms in the ITS region were base substitutions. The numbers of transitions/transversions by pairwise comparisons of nucleotide substitutions of 14 taxa from *Zea* species and the outgroup, *T. dactyloides*, were shown in Table 4. There were only a few base pair differences between *Z. luxurians* and *Z. nicaraguensis*. *Z. luxurians* (Tz06) and *Z. nicaraguensis* (Tz10) had only 1-bp transition, and *Z. luxurians* (Tz05) and Tz10 had only 1-bp transversion, indicating their identical ITS sequences. The similarity in ITS sequences between the two species was expected since analysis of C-banding patterns [8] have suggested that they are apparently closely related.

**Table 4:** Direct counts of transitions/transversions of pairwise comparisons.

	Tz01	Tz02	Tz03	Tz04	Tz05	Tz06	Tz07	Tz08	Tz09	Tz10	Tz11	Tz12	Tz13	Tz14	Trip
Tz01	—														
Tz02	6/1	—													
Tz03	5/2	10/1	—												
Tz04	4/2	8/1	1/0	—											
Tz05	2/3	6/2	3/5	2/4	—										
Tz06	3/2	7/1	4/3	3/2	1/1	—									
Tz07	5/5	9/4	2/4	1/3	3/6	4/5	—								
Tz08	5/4	9/3	2/4	1/3	3/5	4/4	2/3	—							
Tz09	3/2	7/1	6/2	5/2	3/4	4/3	6/6	6/5	—						
Tz10	2/2	6/1	3/4	2/3	0/1	1/0	3/5	3/4	3/3	—					
Tz11	5/3	9/2	2/3	1/2	3/6	4/5	2/5	2/6	6/4	3/5	—				
Tz12	4/5	8/4	1/3	0/3	2/7	3/5	1/1	1/4	5/5	2/6	1/5	—			
Tz13	7/6	11/5	4/7	3/6	5/8	6/7	4/7	4/5	8/7	5/7	4/9	3/8	—		
Tz14	4/4	8/3	1/4	0/3	2/6	3/4	1/1	1/3	5/5	2/4	2/4	0/2	3/7	—	
Trip	19/26	23/25	20/27	19/26	17/23	18/24	20/29	20/27	20/27	17/24	21/27	19/29	22/29	19/28	—

### Phylogenetic inference based on ITS data

**Figure 4:**
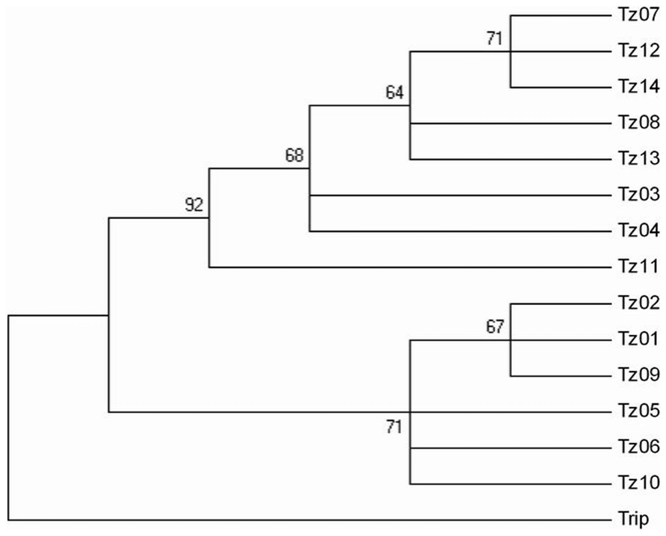
A phylogenetic tree based on neighbor-joining (NJ) using ITS data from 14 taxa of *Zea* and the outgroup taxa of *Tripsacum* (Trip). Numbers above branches indicate bootstrap values from 1000 replications.

Treating gaps as missing data, the phylogenetic trees of 14 taxa in *Zea* based on ITS sequence variation were constructed, and rooted by *Tripsacum* species (Trip). As shown in the NJ (neighbor-joining) tree (Figure 4), 14 samples of *Zea* were also grouped into two major clusters similar to the result of RAPD analysis, among which the taxa from section *Zea* composed a cluster supported with a high bootstrap support (BS) of 92%, and the taxa from section *Luxuriantes* including *Z. nicaraguensis* were all members of the other cluster (BS = 71%). The topology of the MP (maximum parsimony) tree was almost identical to that of the NJ tree, except for a few minor differences at the bootstrap values of the branches (results not shown). Several subclades appeared further within the two major clusters in the NJ tree. The subspecies of *Z. mays* clade formed sister groups with bootstrap support of about 70% which were distinct from the outgroup. *Z. diploperennis*, together with the two Z. *perennis* species, were in the moderately supported group (BS = 67%), which was a sister group to *Z. luxurians* and *Z. nicaraguensis*. From the ITS phylogenetic tree (Figure 4), it is apparently reconfirmed that *Z. nicaraguensis* located in section *Luxuriantes* clade had the closest association with *Z. luxurians*.

**Figure 5:**
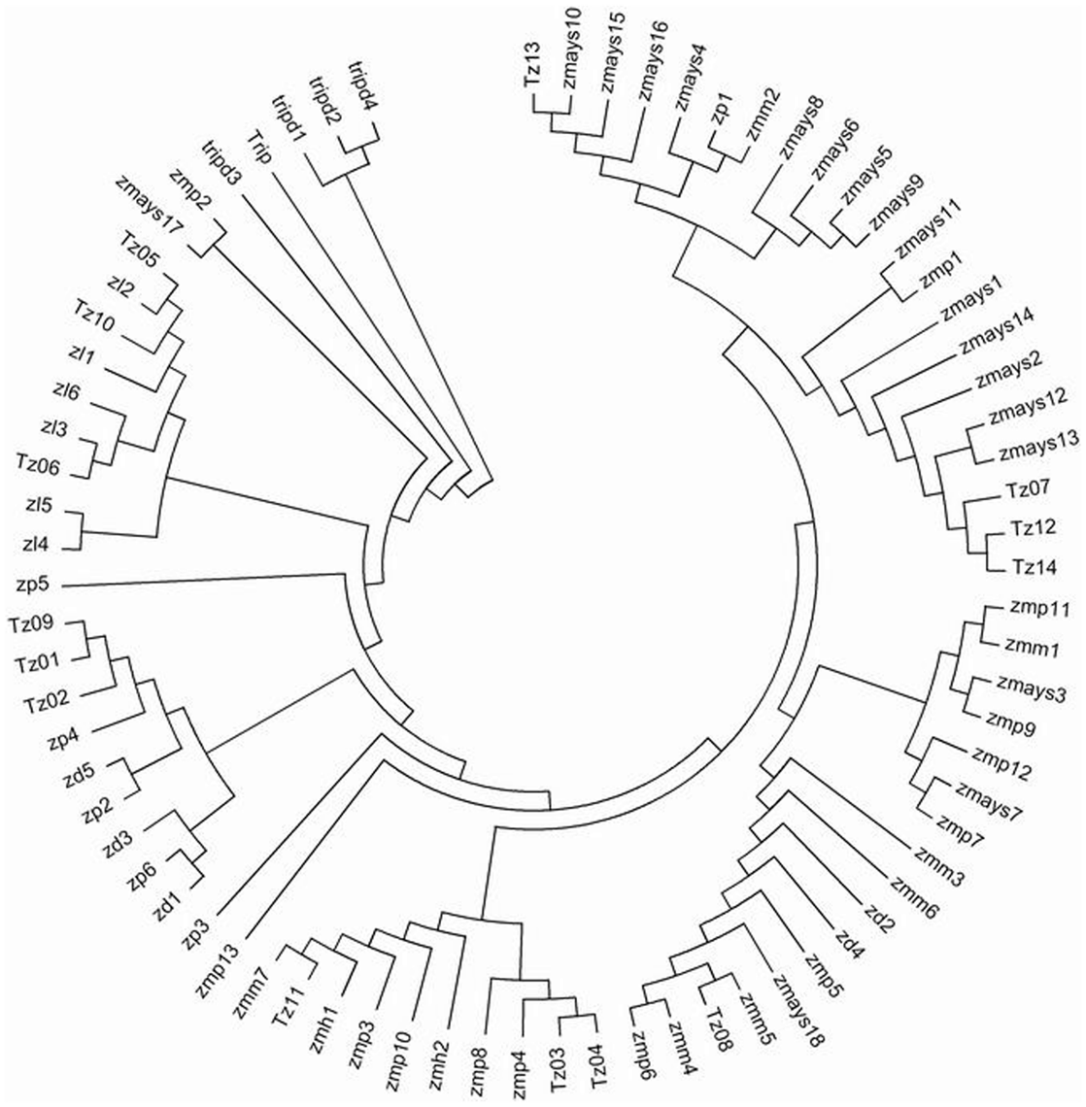
A neighbor-joining tree with the ITS data in the present study and downloaded from GenBank included. zd—*Z. diploperennis*, zp—*Z. perennis*, zl—*Z. luxurians*, zmh—*Z. mays*. ssp. *huehuetenangensis*, zmm—*Z. mays*. ssp. *mexicana*, zmp—*Z. mays*. ssp. *parviglumis*, zmays—*Z. mays*. ssp. *mays*, tripd—*Tripsacum dactyloides*.

As defined in Figure 5, the phylogenetic tree reconstructed combining our ITS sequences with data from GenBank (Table 5) was rooted with *Tripsacum* species (including Trip and tripd1- tripd 4). Our ITS sequences, such as *Z. luxurians* (Tz05 and Tz06), *diploperennis* (Tz09), *Z. perennis* (Tz01 and Tz02), *Z. mays* ssp. *huehuetenangensis* (Tz11), *Z. mays* ssp. *mays* (Tz12, Tz13 and Tz14), *Z. mays* ssp. *parviglumis* (Tz03 and Tz04) and *Z. mays* ssp. *mexicana* (Tz08), were grouped completely consistently with the corresponding taxa on the GenBank database. But *Z. mays* ssp. *mexicana* (Tz07) gathered with GenBank sequences from *Z. mays* ssp. *mays* (zmays); GenBank sequences from *Z. mays* ssp. *mexicana* (zmm) and *Z. mays* ssp. *parviglumis* (zmp) didn't form stable monophyletic clades, few of which mingled with *Z. mays* ssp. *mays* (zmays). These may be due to introgression between cultivated maize and the annual teosinte (*Z. mays* ssp. *mexicana* or *Z. mays* ssp. *parviglumis*). The result indicates that the ITS sequences obtained in our laboratory possess high value, phylogenetic analysis of which can reflect genetic relationships of maize and teosinte in *Zea*.

**Table 5:** GenBank sequences used in this study.

Species	GenBank accession Nos.	Code name
*Zea perennis*	U46583–U46588	zp1–zp6
*Zea diploperennis*	U46589–U46593	zd1–zd5
*Zea luxurians*	U46594–U46599	zl1–zl6
*Zea mays* ssp. *mays*	U46600–U46617	zmays1–zmays18
*Zea mays* ssp. *parviglumis*	U46618–U46630	zmp1–zmp13
*Zea mays* ssp. *mexicana*	U46638–U46644	zmm1–zmm7
*Zea mays* ssp. *huehuetenangensis*	U46645–U46646	zmh1–zmh2
*Tripsacum dactyloides* L.	U46649–U46652	tripd1–tripd4

## Discussion

### RAPD-based UPGMA analysis

The subgeneric boundaries of *Zea* proposed by Doebley and Iltis [1], [2] are well defined on UPGMA-based dendrogram derived from RAPD analysis (Figure 2). The members of each section share a suite of morphological and genetic features [3]. Interestingly, the grouping of populations representing the *Zea* species in the subgenus interior clades is more congruent with grow habitats as well as their geographical locations than morphological data according to Wilkes [19], a little bit different with the system of classification proposed by Doebley and Iltis [1], [2]. In our study, there are eight recognized species of teosinte *Z. diploperennis*, *Z. perennis*, *Z. luxurians*, *Z. nicaraguensis*, *Z. mays* ssp. *mexicana*, *Z. mays* ssp. *parviglumis*, *Z. mays* ssp. *huehuetenangensis* and cultivated maize, which can be basically divided into two sections, annual species and perennial species, based on RAPD cluster analysis. The annual species have been then classified as annual teosinte and cultivated maize. The annual teosintes were then further divided into two parts, one part including *Z. mays* ssp. *mexicana*, *Z. mays* ssp. *parviglumis*, and *Z. mays* ssp. *huehuetenangensis*, and the other including *Z. luxurians* and *Z. nicaraguensis*. For the perennial section, it has been classified as *Z. perennis* (2n = 40) and *Z. diploperennis* (2n = 20). In 1967, Wilkes had presented a system of classification for teosinte which furnished the different geographic populations with separate racial designations [3], [19]. As treated by Wilkes [19], *Zea* is divided into two sections: section *Eucblaena* comprising *Zea perennis* and *Zea mexicana* which includes races Chalco Central Plateau, Nobogame, Balsas and Huehuetenango, and section *Zea* consisting of *Zea mays* L. only. The subspecific taxa of teosinte, like some of Wilkes' races, are differentiated by traits which can vary widely depending upon the conditions of growth.

### ITS variation

The high GC content and secondary structure were presented in this study for the ITS region of *Zea*. We used high success-rate DNA polymerase KOD FX to circumvent these PCR amplification problems. KOD FX is effective for the amplification of GC-rich targets and crude samples, and the PCR error ratio is about 10 times less than that of Taq DNA polymerase. Considering the conditions used here, the error rate should be less than 0.02 bases per ITS clone [16]. Therefore, the nucleotide variation in *Zea* ribosomal ITS sequences was hardly a PCR artifact.

The level of genetic variation observed in our ITS data set (0.17–2.92% sequence divergence among taxa of *Zea*, only 10 informative sites) was compared with ITS variation in other angiosperms studied. ITS sequence divergence ranged from 0.4 to 15.6% and 247 sites were informative within the alpine *Saxifraga*, sect. *Ligulatae* (Saxifragaceae) [20], and the corresponding values were 0.5–17% and 238 in *Iris* series *Californicae* (Iridaceae) of the North American Pacific Coast [21]. It was shown that our intrageneric variation of ITS sequences was much lower than those in these angiosperms. However, the ITS variation value and the number of informative positions in the present data set were comparable to those found in some other angiosperms. For example, 0–1.48% sequence divergence and 10 informative characters were recorded in the *Oxytropis campestris* and *O. arctica* (Fabaceae) complexes in Alaska [15]; pairwise nucleotide sequence differences within the European montane/alpine endemic *Soldanella* (Primulaceae) ranged from 0 to 2.1%, with a mean value of 1.0% [22]; only 9 informative characters were detected in the circumboreal species *Saxifraga oppositifolia* and some related taxa [23].

### ITS phylogeny of the *Zea*

ITS phylogeny depicted two major groups of the genus *Zea*. These two groups are distinct and consistent with the RAPD tree, which are supported by classification of *Zea* according to Doebley and Iltis [1], [2]. The phylogenetic tree of *Zea* (Figure 4) inferred from the ITS sequences shows that each subgenus is monophyletic. When *Tripsacum* was used as an outgroup, the taxa in section *Luxuriantes* were consistently placed as the sister groups, and albeit with not very strong support, they were close to each other in space position at a major cluster located on the base of the phylogenetic tree and clearly differentiated from the monophyletic *Z. mays* clade. The moderate bootstrap values for interior clades suggest that there is abundant sequence polymorphism or introgression among species or subspecies in *Zea* [16]. Figure 5 indicates that our ITS data largely correspond to the previously published ITS sequences of *Zea*, although there are a few samples that are not always well grouped. Our ITS data favor previous phylogenies based on chloroplast and isozyme evidence, which significantly supported a *Z. luxurians*, *Z. diploperennis* and *Z. perennis* clade [3], [24], [25] and a *Z. mays* clade. *Z. perennis* and *Z. diploperennis* are essentially indistinguishable, supported by previous reports from rDNA sequences [16] and microsatellites [26], probably reflecting a recent divergence. The ribosomal ITS relationships between *Z. mays*. ssp. *parviglumis*, *Z. mays*. ssp. *mexicana*, *Z. mays*. ssp. *huehuetenangensis* and *Z. mays*. ssp. *mays* are complicated by the recent divergence of these taxa [16]. The present result is corroborated by microsatellite evidence [26] of admixture between *Z. mays* ssp. *mays* and the Mexican annual teosintes (ssp. *mexicana* and ssp. *parviglumis*) (Figure 5); however, it may also be a function of the recent divergence between these taxa such that their gene pools are not yet completely differentiated. A likely explanation is that maize and annual teosinte are very close geographically and cytogenetically, and except for rare chromosomal rearrangements, their basic genomes are essentially homologous [27]. Another explanation is probably genetic exchange between conventional maize landraces and teosinte owing to crossability among them [28]–[30]. Fukunaga et al. [26] identified 56 out of 117 teosinte plants containing 20% or more of maize germplasm. In their opinion, teosinte grows near maize in most locations and is capable of hybridizing with maize, allowing admixture between maize and the teosintes to occur [26]. Gene flow among maize (*Zea mays* ssp. *mays*) and teosinte (*Zea* spp.) populations has occurred readily since maize's domestication 9,000 years ago [31]–[33]. In spite of crossability between maize and teosinte, more studies suggest that gene flow is mainly unidirectional from teosintes to maize, with either insignificant introgression from maize to teosintes or none at all in either direction [25], [34]. For example, the *Teosinte crossing barrier-1* (*Tcb1-s*) or *Gametophyte–1* (*Ga1*) incompatibility allele in teosinte can prevent crossing in the direction of maize to teosinte [35], [36]. However, gene flow or introgression among different teosinte types has not been studied thoroughly until now, and the genetic affinities between maize and teosinte need to be better understood. Furthermore, the phylogenetic position of *Z. nicaraguensis*, a newly discovered teosinte, in *Zea* species should be further investigated systematically. The results of the present study indicate that it has been clearly defined as being a taxon of section *Luxuriantes* (Figures 4–5).

### Relationship of *Z. nicaraguensis* in *Zea*

As expected, both RAPD cluster and ITS sequence analyses showed that the new teosinte species found on the Pacific cost of Nicaragua, *Z. nicaraguensis*, was clearly classified into section *Luxuriantes*, which was most closely allied to *Z. luxurians*. *Z. luxurians*, an annual with similar, strongly two-nerved lower glumes, is known mostly from a restricted region of southeastern Guatemala, although it has outlier populations in Honduras and Nicaragua [5]. Both *Z. nicaraguensis* and *Z. luxurians* possess unique flooding-related traits such as the capacity to form root aerenchyma under non-flooding conditions and the ability to form adventitious roots at the soil surface under flooding conditions [37]. SSR data confirmed that *Z. nicaraguensis* was not strongly differentiated from *Z. luxurians* [26]. The similarity in C-banding pattern most likely reflected a closer relationship between *Z. nicaraguensis* and *Z. luxurians* [8]. This has been confirmed by the highest RAPD genetic similarity and the lowest pairwise nucleotide sequence divergence between the two species as revealed in this study. Whether or not *Z. nicaraguensis* and *Z. luxurians* should be regarded as subspecies or separate species still needs to be considered with the cytological meiosis evidence. Fukunaga et al. [26] proposed that the status of *Z. nicaraguensis* should be investigated by determining its cross compatibility with *Z. luxurians*. If they are interfertile, then it would be best to treat *Z. nicaraguensis* as a subspecies of *Z. luxurians*. Cultivated maize (*Z. mays* ssp. *mays*) has mostly subterminal knobs and only a few terminal ones, which makes it different from the wild teosinte species in section *Luxuriantes*. All chromosomes of *Z. nicaraguensis* except for chromosome 10 have terminal knobs [8], demonstrating that *Z. nicaraguensis* is more similar to section *Luxuriantes* species than to maize. However, no published reports of natural or artificial hybridization between the other teosintes and the Nicaraguan teosinte *Z. nicaraguensis* are known.

### Conclusions

In conclusion, the genetic relationships among *Zea* species were reported in many previous studies on the basis of morphological, cytogenetical, chemical, and molecular data [4]. The present work provided the first molecular evidence for systematic assessment of genetic relationship of *Z. nicaraguensis* in *Zea* species. The results of the analyses using two types of genetic markers, RAPD and ITS, indicate that *Z. nicaraguensis* has been clearly defined as being the taxon of section *Luxuriantes*. The Guatemalan teosinte *Z. luxurians* is the closest relative to the Nicaraguan teosinte *Z. nicaraguensis*.

## Materials and Methods

### Plant materials and DNA extraction

The plant materials used in the present study are listed in Table 6, which were obtained from the International Maize and Wheat Improvement Center (CIMMYT) and United States Department of Agriculture (USDA). *Tripsacum dactyloides* L. (2n = 72, sample No. was Trip) from USA was selected as the outgroup for ITS sequence analysis according to the phylogenetics of *Zea* and *Tripsacum* reported by Buckler and Holtsford [16]. Total DNA was extracted from young leaves of each of the genotypes using a CTAB method (modified after Saghai-Maroof et al. [38]).

**Table 6:** Species accession name/chromosome number and source of the plant materials used in the study.

Sample No.	Scientific name	Source	Accession	Chromosome number
Tz01	*Zea perennis*	CIMMYT	9475	2n = 40
Tz02	*Zea perennis*	USDA	Ames 21785	2n = 40
Tz03	*Zea mays* ssp. *parviglumis*	CIMMYT	8774	2n = 20
Tz04	*Zea mays* ssp. *parviglumis*	USDA	PI 621785	2n = 20
Tz05	*Zea luxurians*	CIMMYT	9478	2n = 20
Tz06	*Zea luxurians*	USDA	PI 441933	2n = 20
Tz07	*Zea mays* ssp. *mexicana*	CIMMYT	1l394	2n = 20
Tz08	*Zea mays* ssp. *mexicana*	USDA	Ames 8083	2n = 20
Tz09	*Zea diploperennis*	CIMMYT	10003	2n = 20
Tz10	*Zea nicaraguensis*	USDA	PI 615697	2n = 20
Tz11	*Zea mays* ssp. *huehuetenangensis*	USDA	PI 441934	2n = 20
Tz12	*Zea mays* ssp. *mays*	China	48-2	2n = 20
Tz13	*Zea mays* ssp. *mays*	China	08	2n = 20
Tz14	*Zea mays* ssp. *mays*	USDA	Mol7	2n = 20

### RAPD and ITS sequences amplifications

RAPD reactions were performed using 136 random decamer primers with stable and readily reproducible banding patterns screened from a total of 340 primers purchased from Beijing SBS Genetech Co., Ltd (for sequences of the 136 primers, see Table S1). Amplification conditions were 25 µL reactions with ddH_2_O 16.8 µL, 10×PCR buffer 2.5 µL, MgC1_2_ (25 mmol/L) 1.5 µL, dNTPs (10 mmol/L) 2.0 µL, primer (10 pmo1/µL) 1 µL, Taq polymerase (5 U/µL) 0.2 µL, and genomic DNA (50 ng/µL) 1 µL. The PCR parameters were 94°C for 5 min, 38 cycles of 94°C for 1 min, 38°C for 1 min, and 72°C for 1.5 min, followed by a final extension step at 72°C for 7 min. Amplification products were separated by size on 1.5% agarose gels, stained with ethidium bromide (EB), and visualized under UV (ultraviolet) light. Pictures were taken with an Electrophoresis Systems Photo Documentation Camera.

The ITS region (including ITS1, 5.8S and ITS2) of each sample was amplified with primers P1: 5′-TCGTAACAAGGTTTCCGTAGG-3′ and P4: 5′-TCCTCCGCTTATTGATATGC-3′ [39], synthesized by Shanghai Invitrogen Biotechnology Co., Ltd. The DNA fragment amplified using these two primers is approximately 700 bp long. Amplification conditions were 25 µL reactions by adding 12.5 µL 2×PCR buffer for KOD FX, 4.5 µL ddH_2_O, 5.0 µL dNTPs (2 mmol/L), 0.75 µL P1 (10 pmo1/µL), 0.75 µL P4 (10 pmo1/µL), 0.5 µL KOD FX (1 U/µL) (Toyobo Co., Ltd.), and 1 µL genomic DNA (50 ng/µL). The PCR parameters were 94°C for 4 min, 38 cycles of 94°C for 1 min, 61°C for 1 min, and 72°C for 1.5 min, followed by a final extension step at 72°C for 8 min. The PCR products were separated on 1.5% agarose gels and visualized with EB. Due to the smooth terminal of target bands amplified by using high fidelity PCR enzyme KOD FX, it was necessary to add “A” tail to the terminal of the PCR products before TA cloning. Then PCR products were purified with TIANgel Midi Purification Kit (TIANGEN, Beijing, China) following the protocol from the manufacturer, and ligated into a pMD19-T vector (TaKaRa, Dalian, China). Both gel-purified PCR products and cloned inserts which were confirmed in the range of about 700 bp were used as templates for sequencing. Difficulties with obtaining sequence information from PCR-amplified products from some samples made it necessary to derive the complete sequence from cloned templates. In this case, at least two different clones were sequenced to minimize the impact of errors caused by PCR amplification. Sequencing was conducted on an ABI 3730 automated sequencer (Invitrogen, Shanghai, China).

### Analysis of RAPD and ITS data

Photographs were used to score the data for RAPD analysis, and DNA fragment sizes were estimated by comparisons with DNA size markers run on the same gel. RAPD bands were scored as present (1) or absent (0) in each genotype for each set of primers to create binary data matrices and only strong, clear and reproducible bands were used in this study. The data matrices were entered into the NTSYS-pc2.10 software and pairwise distance matrices were computed based on Dice similarity coefficients using the SIMQUAL (similarity for qualitative data) routine [40]. Similarity coefficients were used to construct the UPGMA (unweighted pair group method with arithmetic average) dendrograms [41] using the SHAN (sequential, hierarchical, agglomerative, and nested cluster methods) clustering and a tree was displayed through the tree plot routine in the software.

Sequences of the entire ITS region from 14 *Zea* species together with that of the outgroup species were used to determine ITS1, 5.8S and ITS2 boundaries by homologous blast with published ITS sequences on the GenBank database, and were subsequently aligned with the Clustal X program [42]. Then, phylogenetic analyses of the aligned ITS sequences (the entire ITS1-5.8S-ITS2 region) was performed in MEGA 4.0 (Molecular Evolutionary Genetics Analysis, version 4.0) to calculate the characteristics of the ITS regions and to construct the phylogenetic trees employing neighbor-joining (NJ) and maximum parsimony (MP) methods. Calculations were made after considering gaps as missing data [22]. A preliminary analysis including all sequenced clones and PCR products was conducted. If all the clones from one sample were supported as monophyletic or as components of the same polytomy, a representative sequence showing the fewest autapomorphies was chosen for all further analyses. MEGA 4.0 was also used to calculate the proportion of nucleotide sequence differences (adjusted for missing data and gaps), and Kimura two-parameter distances as the measure of sequence divergence. In addition, bootstrap analysis (1000 replications) was performed to obtain estimates of support for clades of the ITS trees. To verify our ITS data further, GenBank data were used in sequence analysis together with the ITS sequences determined in our laboratory, all of which came from studies by Buckler and Holtsford [16] (Table 5). Four GenBank sequences from species within *Tripsacum* (*Tripsacum dactyloides*) were used as the outgroup for phylogenetic analysis.

## Supporting Information

Table S1 Sequences of 136 RAPD primers used in PCR amplification.
(DOC)
