# Supplementary material for: RAPD and Internal Transcribed Spacer Sequence Analyses Reveal Zea nicaraguensis as a Section Luxuriantes Species Close to Zea luxurians
Source: PLoS One. 2011 Apr 15;6(4):e16728. doi: 10.1371/journal.pone.0016728 (PMC3078115; doi:10.1371/journal.pone.0016728)
Supplement: Table S1 — Sequences of 136 RAPD primers used in PCR amplification. (DOC) [file pone.0016728.s001.doc]

Table S1 Sequences of 136 RAPD primers used in PCR amplification.

| Primers | Sequence | Primers | Sequence | Primers | Sequence | Primers | Sequence |
| --- | --- | --- | --- | --- | --- | --- | --- |
| A01 | 5'-CAGGCCCTTC-3' | F08 | 5'-GGGATATCGG-3' | J20 | 5'-AAGCGGCCTC-3' | M18 | 5'-CACCATCCGT-3' |
| A02 | 5'-TGCCGAGCTG-3' | G02 | 5'-GGCACTGAGG-3' | K01 | 5'-CATTCGAGCC-3' | M19 | 5'-CCTTCAGGCA-3' |
| A09 | 5'-GGGTAACGCC-3' | G03 | 5'-GAGCCCTCCA-3' | K03 | 5'-CCAGCTTAGG-3' | N01 | 5'-CTCACGTTGG-3' |
| A10 | 5'-GTGATCGCAG-3' | G04 | 5'-AGCGTGTCTG-3' | K04 | 5'-CCGCCCAAAC-3' | N02 | 5'-ACCAGGGGCA-3' |
| A11 | 5'-CAATCGCCGT-3' | G07 | 5'-GAACCTGCGG-3' | K08 | 5'-GAACACTGGG-3' | N03 | 5'-GGTACTCCCC-3' |
| A20 | 5'-GTTGCGATCC-3' | G08 | 5'-TCACGTCCAC-3' | K10 | 5'-GTGCAACGTG-3' | N04 | 5'-GACCGACCCA-3' |
| B05 | 5'-TGCGCCCTTC-3' | G16 | 5'-AGCGTCCTCC-3' | K12 | 5'-TGGCCCTCAC-3' | N11 | 5'-TCGCCGCAAA-3' |
| B06 | 5'-TGCTCTGCCC-3' | G18 | 5'-GGCTCATGTG-3' | K13 | 5'-GGTTGTACCC-3' | N15 | 5'-CAGCGACTGT-3' |
| B07 | 5'-GGTGACGCAG-3' | G19 | 5'-GTCAGGGCAA-3' | K14 | 5'-CCCGCTACAC-3' | N19 | 5'-GTCCGTACTG-3' |
| B08 | 5'-GTCCACACGG-3' | H02 | 5'-TCGGACGTGA-3' | K15 | 5'-CTCCTGCCAA-3' | N20 | 5'-GGTGCTCCGT-3' |
| B10 | 5'-CTGCTGGGAC-3' | H03 | 5'-AGACGTCCAC-3' | K16 | 5'-GAGCGTCGAA-3' | O02 | 5'-ACGTAGCGTC-3' |
| B12 | 5'-CCTTGACGCA-3' | H05 | 5'-AGTCGTCCCC-3' | K17 | 5'-CCCAGCTGTG-3' | O04 | 5'-AAGTCCGCTC-3' |
| B13 | 5'-TTCCCCCGCT-3' | H07 | 5'-CTGCATCGTG-3' | K19 | 5'-CACAGGCGGA-3' | O06 | 5'-CCACGGGAAG-3' |
| B14 | 5'-TCCGCTCTGG-3' | H08 | 5'-GAAACACCCC-3' | K20 | 5'-GTGTCGCGAG-3' | O07 | 5'-CAGCACTGAC-3' |
| B20 | 5'-GGACCCTTAC-3' | H09 | 5'-TGTAGCTGGG-3' | L02 | 5'-TGGGCGTCAA-3' | O11 | 5'-GACAGGAGGT-3' |
| C01 | 5'-TTCGAGCCAG-3' | H12 | 5'-ACGCGCATGT-3' | L03 | 5'-CCAGCAGCTT-3' | O12 | 5'-CAGTGCTGTG-3' |
| C04 | 5'-CCGCATCTAC-3' | H13 | 5'-GACGCCACAC-3' | L04 | 5'-GACTGCACAC-3' | O13 | 5'-GTCAGAGTCC-3' |
| C06 | 5'-GAACGGACTC-3' | H14 | 5'-ACCAGGTTGG-3' | L05 | 5'-ACGCAGGCAC-3' | O15 | 5'-TGGCGTCCTT-3' |
| C07 | 5'-GTCCCGACGA-3' | H16 | 5'-TCTCAGCTGG-3' | L07 | 5'-AGGCGGGAAC-3' | O16 | 5'-TCGGCGGTTC-3' |
| C09 | 5'-CTCACCGTCC-3' | H20 | 5'-GGGAGACATC-3' | L08 | 5'-AGCAGGTGGA-3' | O19 | 5'-GGTGCACGTT-3' |
| C11 | 5'-AAAGCTGCGG-3' | I01 | 5'-ACCTGGACAC-3' | L11 | 5'-ACGATGAGCC-3' | O20 | 5'-ACACACGCTG-3' |
| C12 | 5'-CACCGTATCC-3' | I02 | 5'-GGAGGAGAGG-3' | L12 | 5'-GGGCGGTACT-3' | P01 | 5'-GTAGCACTCC-3' |
| D01 | 5'-ACCGCGAAGG-3' | I04 | 5'-CCGCCTAGTC-3' | L13 | 5'-ACCGCCTGCT-3' | P03 | 5'-CTGATACGCC-3' |
| D02 | 5'-GGACCCAACC-3' | I10 | 5'-ACAAGGCGAG-3' | L14 | 5'-GTGACAGGCT-3' | P04 | 5'-GTGTCTCAGG-3' |
| D03 | 5'-GTCGCCGTCA-3' | I11 | 5'-ACATGCCGTG-3' | L15 | 5'-AAGAGAGGGG-3' | P08 | 5'-ACATCGCCCA-3' |
| E08 | 5'-TCACCACGGT-3' | I13 | 5'-CTGGGGCTGA-3' | L16 | 5'-AGGTTGCAGG-3' | P09 | 5'-GTGGTCCGCA-3' |
| E09 | 5'-CTTCACCCGA-3' | I15 | 5'-TCATCCGAGG-3' | M02 | 5'-ACAACGCCTC-3' | Q01 | 5'-GGGACGATGG-3' |
| E10 | 5'-CACCAGGTGA-3' | I16 | 5'-TCTCCGCCCT-3' | M06 | 5'-CTGGGCAACT-3' | Q06 | 5'-GAGCGCCTTG-3' |
| E11 | 5'-GAGTCTCAGG-3' | J01 | 5'-CCCGGCATAA-3' | M09 | 5'-GTCTTGCGGA-3' | Q09 | 5'-GGCTAACCGA-3' |
| E17 | 5'-CTACTGCCGT-3' | J04 | 5'-CCGAACACGG-3' | M11 | 5'-GTCCACTGTG-3' | Q10 | 5'-TGTGCCCGAA-3' |
| E19 | 5'-ACGGCGTATG-3' | J09 | 5'-TGAGCCTCAC-3' | M14 | 5'-AGGGTCGTTC-3' | Q12 | 5'-AGTAGGGCAC-3' |
| F05 | 5'-CCGAATTCCC-3' | J17 | 5'-ACGCCAGTTC-3' | M15 | 5'-GACCTACCAC-3' | Q14 | 5'-GGACGCTTCA-3' |
| F06 | 5'-GGGAATTCGG-3' | J18 | 5'-TGGTCGCAGA-3' | M16 | 5'-GTAACCAGCC-3' | Q15 | 5'-GGGTAACGTG-3' |
| F07 | 5'-CCGATATCCC-3' | J19 | 5'-GGACACCACT-3' | M17 | 5'-TCAGTCCGGG-3' | Q17 | 5'-GAAGCCCTTG-3' |
